# GACT: a Genome build and Allele definition Conversion Tool for SNP imputation and meta-analysis in genetic association studies

**DOI:** 10.1186/1471-2164-15-610

**Published:** 2014-07-19

**Authors:** Arvis Sulovari, Dawei Li

**Affiliations:** 1 Department of Microbiology and Molecular Genetics, University of Vermont, 05405 Burlington, VT, USA; 2 Cell, Molecular and Biomedical Sciences Graduate Program, University of Vermont, 05405 Burlington, VT, USA; 3 Department of Computer Science, University of Vermont, 05405 Burlington, VT, USA; 4 Neuroscience, Behavior and Health Initiative, University of Vermont, 05405 Burlington, VT, USA

**Keywords:** Allele definition (nomenclature), Genome build, Genome-wide association study (GWAS), Imputation, Meta-analysis

## Abstract

**Background:**

Genome-wide association studies (GWAS) have successfully identified genes associated with complex human diseases. Although much of the heritability remains unexplained, combining single nucleotide polymorphism (SNP) genotypes from multiple studies for meta-analysis will increase the statistical power to identify new disease-associated variants. Meta-analysis requires same allele definition (nomenclature) and genome build among individual studies. Similarly, imputation, commonly-used prior to meta-analysis, requires the same consistency. However, the genotypes from various GWAS are generated using different genotyping platforms, arrays or SNP-calling approaches, resulting in use of different genome builds and allele definitions. Incorrect assumptions of identical allele definition among combined GWAS lead to a large portion of discarded genotypes or incorrect association findings. There is no published tool that predicts and converts among all major allele definitions.

**Results:**

In this study, we have developed a tool, GACT, which stands for **G**enome build and **A**llele definition **C**onversion **T**ool, that predicts and inter-converts between any of the common SNP allele definitions and between the major genome builds. In addition, we assessed several factors that may affect imputation quality, and our results indicated that inclusion of singletons in the reference had detrimental effects while ambiguous SNPs had no measurable effect. Unexpectedly, exclusion of genotypes with missing rate > 0.001 (40% of study SNPs) showed no significant decrease of imputation quality (even significantly higher when compared to the imputation with singletons in the reference), especially for rare SNPs.

**Conclusion:**

GACT is a new, powerful, and user-friendly tool with both command-line and interactive online versions that can accurately predict, and convert between any of the common allele definitions and between genome builds for genome-wide meta-analysis and imputation of genotypes from SNP-arrays or deep-sequencing, particularly for data from the dbGaP and other public databases.

**GACT software:**

http://www.uvm.edu/genomics/software/gact

## Background

Genome-wide association studies (GWASs) and next-generation deep sequencing studies have successfully identified genes associated with human diseases and traits, yet they suggest that the identified variants cumulatively explain a small percentage of the estimated inherited risk to develop these diseases. Combining samples from multiple GWASs or deep sequencing datasets of the same phenotype for large-scale meta-analyses will increase the statistical power to identify new or rare associated variants [1], particularly for complex traits where the disease variants may have moderate effect sizes, which may account for some of the missing heritability [2]. However, the raw single nucleotide polymorphism (SNP) genotype datasets might have been generated using different genotyping or sequencing platforms, array types [3] or SNP calling procedures, resulting in the use of different genome builds or allele definitions (nomenclatures). Thus, combining multiple GWASs or deep sequencing studies (e.g. the 1000 Genomes Project [4]) requires conversions of inconsistent allele definitions and genome builds between the datasets, as demonstrated in a large number of NHGRI (http://www.genome.gov) GWAS meta-analyses [1]. Likewise, imputation, one of the commonly-used approaches to predict the genotypes for un-assayed loci, requires the same consistency between the study and reference datasets, for example, imputation has been applied to almost half of the GWASs [1] in the NHGRI GWAS Catalog.

Four common nomenclatures exist for reporting biallelic SNPs, including: probe/target or A/B, Plus (+)/Minus (−), TOP/BOT, and Forward/Reverse [5]. The genotype data from different studies are often not consistent or matched for genome builds or allele definitions, and thus, genotype and build conversions are required if an investigator combines multiple GWASs or imputes a reference dataset (e.g., the 1000 Genome data) into a study GWAS. For example, different genome builds, primarily build 36 (b36) and b37, and various allele definitions were adopted in the 15,541 NHGRI GWAS Catalog datasets. The solutions that disregard mismatched SNPs, i.e., direct allele-flipping or removal of mismatches [6], will lead to undesirable consequences. For example, allele-flip (i.e., from A1 to A2 and vice versa) ignores the allele frequencies of study population and may make the downstream analyses of the flipped SNPs irrelevant to the sample population; and genotype removal may significantly lower the SNP density of relevant regions. Thus, the build of the human genome that was used to call the study SNPs (or true-genotypes) and the allele definition have to be determined and converted where necessary prior to imputation and meta-analysis.

**Figure 1:**
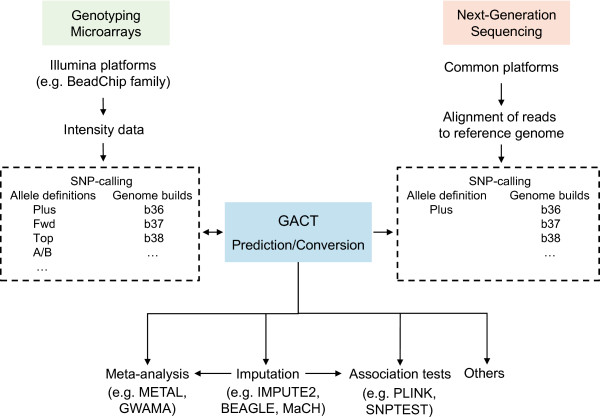
**Study design and GACT functionality.** The left side of the figure indicates that microarray data can be used to call SNPs in any of the four listed SNP definitions. Often, when genotypes are obtained from public repositories (e.g. dbGaP), allele definitions may not be immediately known to investigators. GACT will predict allele definition and genome build, and convert to any new definitions or builds. Since the SNP definition in the NGS data is determined during alignment to the human reference genome (Plus is a commonly-used definition), the SNP alleles from genotyping microarrays can be converted and matched to those from NGS. After GACT’s conversion, imputation, meta-analysis and (or) other analyses may be carried out using the commonly-used tools such as GWAMA, METAL, PLINK, and IMPUTE2.

**Figure 2:**
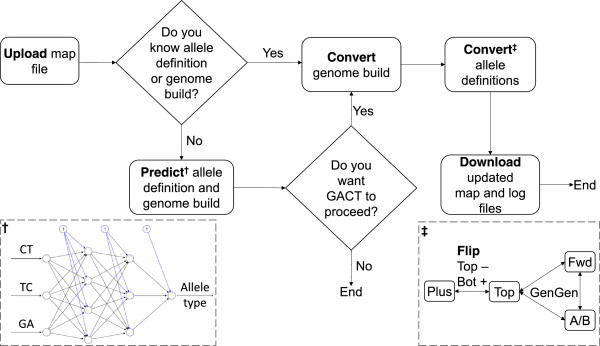
**GACT pipeline.** The flow diagram shows the major procedures in the GACT design. The bottom left panel shows the prediction model of allele definitions based on the distribution of each definition (Figure 2). The bottom right panel shows the allele conversion pathway among the four allele definitions. The input file to be uploaded is a PLINK format map file. This pipeline is implemented in both command-line and web interface.

To our knowledge, there is no available tool that simultaneously predicts and converts human genome builds and allele definitions. The existing tools either convert between selected allele definitions alone (such as GenGen (http://www.openbioinformatics.org/gengen) where the Plus (+)/Minus (−) definition is not included) or between genome builds alone (such as the UCSC Genome Browser LiftOver (genome.ucsc.edu/cgi-bin/hgLiftOver)). In this study, we have developed a new and powerful genotype conversion tool, GACT, which stands for **G**enome build and **A**llele definition **C**onversion **T**ool, to aid in imputation, meta-analysis or both (Figure 1). GACT (Figure 2) directly inter-converts among any of the four allele definitions and between the b36 and b37 genome builds. Since investigators who use datasets from existing GWAS repositories, such as the dbGaP, may not immediately know what allele definitions were used to call the SNPs, we built an artificial neural network (ANN) within GACT to predict the allele definitions. For next-generation sequencing (NGS) projects, since the sequence reads are aligned and mapped to the human reference genome, which is often in the Plus (+)/Minus (−) definition, the SNP genotypes will be of the same one definition. GACT can convert and match the SNP data from genotyping arrays to NGS data (SNP calls) for data merge and meta-analyses. Our example conversions from A/B definition b36 to Plus/Minus definition b37 consistently yielded high matches with the phased 1000 Genomes genotypes (Table 1), demonstrating the accuracy of our tool for converting the genome builds and allele definitions. GACT can be used as a powerful command line application as well as a user-friendly interactive web tool.

**Table 1:** Genotype mismatches between the GWAS and 1000 genomes datasets

**Study GWAS**	**1000 genomes**	**Types**	**Incorrect conversions**		**Correct conversion**
			**Fwd-plus**	**Top-plus**	**Plus-plus**
T/C	C/T	FLIP	0	0	0
T/C	A/G	CSF	5,048	9,875	301
T/C	G/A	FLIP & CSF	8,556	27,648	1,840
T/A	*/*	AMBIG	432	432	432
*/*	−/−	NAR	3,344	3,344	3,344
Matches (%)			62,793 (78.3) (81.7)^†^	38,875 (48.5)	74,256 (92.6) (96.7)^†^

FLIP: switch both alleles with one another (from A1 to A2 and vice versa).

CSF: complimentary strand flip.

AMBIG: ambiguous SNPs in study GWAS.

NAR: not available in the reference.

*/*: any genotype.

−/−: missing genotype.

Fwd: Forward/Reverse.

Top: TOP/BOT.

Plus: Plus (+)/Minus (−).

^†^, percentages of matched genotypes after excluding the NAR genotype counts.

Both the “GWAS” (the 3,096 Ashkenazi Jewish samples) and “1000 Genome” columns show the example alleles in the A1/A2 order. The “Type” column indicates the changes required to match the study SNP to the reference. The last three columns refer to numbers of genotype mismatches on chromosome 1 (80,173 SNPs in total). The “Fwd-Plus” and “Top-Plus” columns show the numbers of genotype mismatches between the “Fwd” and “Top” definitions of our GWAS data (we first generated two versions of the same GWAS data: “Fwd” and “Top”) and the “Plus” definition of the 1000 Genome data, respectively, while the “Plus” column refers to the numbers after we converted the GWAS data to “Plus” using GACT. The last row shows the numbers (percentages) of correct genotype matches (e.g., “T/C” and “T/C”) between the GWAS and 1000 Genome data, where the (%) and (%) ^†^represent the percentages measured by including and excluding the SNPs (NAR) unique to our GWAS data, respectively. Similar ratios were observed in other chromosomes.

Imputation is often desirable before combining multiple genotype datasets from different recourses for meta-analysis. Our imputation analysis revealed higher quality for imputed SNPs when GACT was used, compared to when mismatched SNPs were excluded (Additional file 1: Table S1). While GACT aims to convert between allele definitions and maximize the number of correctly matched alleles to a reference, there are many other factors that can affect imputation quality. Hence, we measured the effects of selected variant types (such as singletons (i.e. SNPs with only one copy of the minor allele among all samples), monomorphic SNPs, and ambiguous SNPs) and GWAS quality control procedures (such as genotype missing rate) on imputation quality. We found that the exclusion of singletons and monomorphic SNPs from the reference improved imputation quality of rare SNPs with minor allele frequency (MAF) < 0.005 (the mean quality score increased from 0.52 to 0.57, which was the highest increase across all MAF ranges) but had no effect on SNPs with MAF > 0.005 (the mean score remained 0.91). The ambiguous SNPs had no measurable effect on imputation, while imputation quality decreased as the genotype missing thresholds became more conservative. Surprisingly, for imputed common SNPs (MAF > 0.1), the decrease in imputation quality started to emerge under very stringent genotype missing thresholds (0.004-0.001, instead of the commonly-used 0.05); by comparison, the imputation of relatively rare SNPs (MAF < 0.1) was even more robust, the decrease was not significant until the missing threshold reached a more stringent threshold of 0.0005 (corresponding to removal of 61.4% of the genotypes). Moreover, the physical locations of the SNPs that were excluded under these missing thresholds were distributed uniformly across the chromosomes. Our analyses provide novel insight into imputation insensitivity to genotype missingness, particularly for rare SNPs.

## Implementation

### Subjects and genotype data

A cohort of 3,096 subjects of Ashkenazi Jewish ethnicity were genotyped using the Illumina Human Omni 1 Quad arrays. The GWAS genotype data were obtained through the NIH dbGaP [phs000448].

### GACT pipeline

GACT was designed for matching allele definitions between the study GWAS and reference data before imputation or merging multiple genome-wide genotype datasets before meta-analysis, where the genotypes were generated from SNP-arrays or deep-sequencing platforms (Figure 1). Figure 2 shows the study design and GACT pipeline, which can be directly connected to other commonly-used methods, including genotype phasing of GWAS (or deep sequencing) data, imputation, data merging, and meta-analysis (Figure 1). The proper execution in command line of GACT requires PLINK [7], GenGen, and the genotyping array annotation files in the same directory, which can be downloaded from our website. The command line follows this syntax (example): *./gact b36 b37 ab plus o1qd map_file_name*. The arguments represent the current genome build (b36), desired genome build (b37), current allele definition (ab), desired allele definition (plus), annotation file of SNP genotyping array (o1qd = Human Omni 1 Quad Duo), and input map file name, respectively. The input file should be in the same format as the PLINK binary map file, containing chromosome location and reference alleles of each SNP. The web version accesses the same command line options on the server-end after user uploads the input file, a PLINK format map file, and chooses the preferred options on the web interface. Moreover, the web tool allows the user to view in real time a log of every step in the conversion process. The command line has no pre-defined limit on the input file size while the web tool has a limit of 40 megabytes (MB), which is sufficient for most SNP arrays (e.g., the entire map file of the Illumina Human Omni 1 Quad array is < 30 MB).

**Figure 3:**
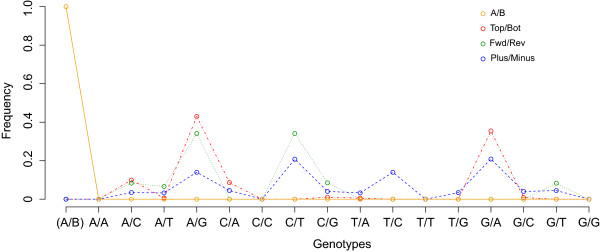
**Frequencies and distributions of all possible genotypes of biallelic SNPs.** The data were generated for the Plus/Minus, Forward/ Reverse, A/B, and TOP/BOT definitions based on the 1000 Genomes, dbSNP, and our GWAS datasets for the last two, respectively. The prediction model of allele definitions was trained using these distributions.

To build the allele definition prediction model, the 1000 Genomes data (2,046,145 SNPs on chromosome 1), dbSNP data (51,864 SNPs on chromosome 1), and our GWAS data (964,554 SNPs on chromosome 1) were used to extract the allele properties of the Plus (+)/Minus (−), Forward/Reverse, and TOP/BOT definitions, respectively (our findings were consistent across all chromosomes). The three genotypes (CT, TC, and GA, Figure 3) that showed the largest amount of differential enrichment among the allele definitions were used as the inputs for a feed-forward, back propagation, ANN with 3 input neurons, 2 hidden layers, and 1 output neuron. This ANN was trained using 10 random samples of various sizes (from 1,000 to 2,000,000 SNPs) from each of the three genotype sources. The ai4r ruby gem (ai4r.org) was used to implement the ANN. Similarly, the coordinates of selected common SNPs in both b36 and b37 datasets were used as the references to predict genome builds. We assessed the quality of implementing our tool to the GWAS data by counting the number of allele matches between the study data and 1000 Genomes Project data using SHAPEIT [8]. GACT was written using a set of Python, Ruby, Hypertext Preprocessor (PHP), and bash scripts. More details and frequently asked questions are available on our website.

### Imputation quality assessment

The GWAS genotype data of the 3,096 Ashkenazi Jewish samples was in b36 genome build and A/B allele definition. GACT was used to convert the allele definition and genome build to the b37 and PLUS allele to keep them consistent with the 1000 Genomes panel. The genotype match rates between the study and reference datasets and imputation quality scores were used as primary measurements to assess conversion quality of GACT. After converting the genome builds and allele definitions in the map files using GACT, we recoded all the genotypes of the GWAS data using PLINK. The genotype phasing and imputation were carried out using SHAPEIT and Impute2 [9], respectively. The latest phased 1000 Genomes genotypes of the European population (Phase 1 integrated release version 3) were used as the imputation reference. Imputation quality was assessed using the Impute2 information scores of the reference SNPs. The scores (equivalent to the r-squared metric reported by MaCH [10] and BEAGLE [11]) vary between 0 and 1, where values closer to 1 represent imputation with high certainty. The mean and standard deviation of these scores were used as measures of overall imputation quality of SNPs at specific MAF ranges. To compare the imputation quality between different MAFs, we used the Welch two sample t-test. All the statistical analyses and graphs were generated using the latest version of R (version 3.0.2), and the imputations were conducted using the multi-core cluster at the Vermont Advanced Computing Center.

## Results

### GACT prediction of genome build and allele definition

We measured the frequencies of all 16 possible genotype patterns under three allele definitions, including Plus (+)/Minus (−), Forward/Reverse, and TOP/BOT (the A/B or probe/target definition is differently coded). The distributions (Figure 3) were clearly distinguishable, and thus used to predict all the four designations. We observed the enrichment of two patterns A/G and G/A, two patterns A/G and C/T, and four patterns A/G, G/A, C/T and T/C for TOP/BOT, Forward/Reverse, and Plus/Minus, respectively. The prediction model matches relative ratios of the input genotypes to the expected ratios in each definition by measuring the proportions of CT, TC and GA alleles present. These three values acted as the input neurons into a multilayer perceptron that classified the input map file into one of the four SNP definitions (Additional file 2: Figure S1). Thus, for users who have no knowledge about the allele definitions and (or) genome build, GACT will first notify the user of the predicted definition and build of the input SNPs prior to actual conversion. The prediction module is particularly useful when the datasets are obtained from public genotype repositories, such as the dbGaP.

### GACT conversion of genome build and allele definition

GACT has been demonstrated to identify and clean all the convertible allele mismatches. Table 1 shows the amounts of genotypes that should be discarded if we incorrectly assumed versus correctly converted the allele definitions between our GWAS data and the 1000 Genome data (Plus/Minus) during imputation. For instance, if we incorrectly converted our GWAS genotypes to the “Forward/Reverse” or “TOP/BOT” definition, and imputed with the 1000 Genome data, we had to discard 21.7% and 51.5% of the genotypes, respectively, due to mismatch. By comparison, if we correctly converted our genotypes to “Plus/Minus” by using GACT, only 7% needed to be discarded across all the chromosomes (Table 1). Moreover, since 3,344 SNPs existed in our data but not in the reference, when only the SNPs that existed in both datasets were used in the calculation, the discarded genotypes only accounted for 3.3%, which was significantly lower than commonly-observed mismatch rates in the literature. The reasons for the 3.3% mismatches are described in the discussion.

**Figure 4:**
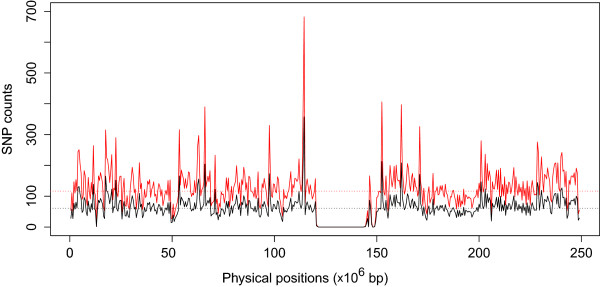
**Comparison of SNP density plots before (“Top” allele definition; black line) and after (“Plus” allele definition; red line) GACT conversion.** The SNP density was measured per 500,000 bp window. It is clear that the SNP count (or density) increase after GACT converts all the mismatched loci, e.g., from 61.05 (median) to 117 SNPs per window. Moreover, it is evident that the increase is not biased with regard to physical location, which indicates that the allele definition mismatches are uniformly distributed across the chromosome. The dotted horizontal lines represent the median of values of each line matched by color. The median, instead of mean, was used since the former was less vulnerable to outliers (e.g. zero counts in the centromere region). The “Forward/Reverse” allele definition showed a similar distribution of mismatches with the 1000 Genomes, however, only the “TOP” definition is shown due to its higher level of mismatches (51.5% mismatches in “TOP” versus 21.7% mismatch in “Forward”). Other chromosomes showed similar patterns, and thus only the results of chromosome 1 are shown.

As expected, the imputation quality decreased when the mismatch rate increased (Additional file 1: Table S1), which was primarily due to the decrease of SNP density in the study data. Figure 4 clearly shows evidence of a significant increase in the SNP density (*P* = 3.2 × 10^−144^ based on 2-sided paired t-test) of the study data across the entire chromosome. Likewise, the imputation quality (information scores) consistently increased by 1% across all MAFs after we converted the genome build and allele definition of our GWAS data from the Forward/Reverse definition (to the Plus/Minus definition) using GACT (Additional file 1: Table S1). However, it should be noted that the improvement would be much higher if we converted the TOP/BOT definition (to the Plus/Minus definition) since without conversions (Table 1) the mismatch rate between the TOP/BOT and Plus/Minus definitions was larger than that between the Forward/Reverse and Plus/Minus definitions.

### Imputation quality

We measured the effects of multiple SNP types and GWAS quality control procedures on imputation quality (i.e., using the information scores). The results (Table 2) showed that the imputation quality increased from 0.52 to 0.57 for the variants with 0.001 < MAF < 0.005 when both the monomorphic variants and singletons were removed from the reference panel, however, no significant change was observed for more common variants with MAF > 0.005. When both of the ambiguous and singleton SNPs were removed from the study data (prior to phasing and imputation), the imputation quality showed no significant changes, which was consistent with previous studies [12].

**Table 2:** Quality scores of the imputed (I) and study (S) SNPs for each MAF category

**Datasets**		**.001-.005**		**.005-.01**		**.01-.05**		**.05-.1**		**.1-.3**		**.3-.5**	
**GWAS**	**1KG**	**I**	**S**	**I**	**S**	**I**	**S**	**I**	**S**	**I**	**S**	**I**	**S**
**All**	**All**	.520 (.222)	.854 (.249)	.727 (.222)	.902 (.184)	.853 (.173)	.945 (.131)	.939 (.118)	.971 (.089)	.965 (.086)	.981 (.060)	.975 (.071)	.981 (.063)
**All**	**NoSin**	.584 (.289)	.854 (.239)	.738 (.227)	.906 (.181)	.855 (.174)	.945 (.132)	.939 (.118)	.970 (.092)	.966 (.086)	.982 (.060)	.975 (.071)	.981 (.064)
**All**	**NoSM**	.571 (.275)	.859 (.245)	.730 (.222)	.901 (.186)	.854 (.172)	.945 (.131)	.939 (.118)	.971 (.089)	.965 (.086)	.981 (.060)	.975 (.071)	.981 (.063)
**NoSin**	**NoSM**	.571 (.275)	.858 (.245)	.730 (.222)	.903 (.184)	.854 (.172)	.945 (.131)	.939 (.118)	.971 (.089)	.965 (.086)	.981 (.060)	.975 (.071)	.981 (.063)
**NoAm**	**NoSM**	.572 (.274)	.855 (.245)	.731 (.222)	.900 (.185)	.854 (.172)	.944 (.131)	.940 (.117)	.971 (.091)	.966 (.085)	.981 (.060)	.975 (.071)	.981 (.064)
**3per**	**NoSM**	.570 (.274)	.859 (.245)	.730 (.222)	.901 (.187)	.853 (.173)	.944 (.131)	.939 (.118)	.970 (.091)	.965 (.086)	.981 (.061)	.974 (.073)	.981 (.064)
**1per**	**NoSM**	.568 (.274)	.851 (.251)	.726 (.223)	.899 (.186)	.851 (.174)	.942 (.134)	.937 (.120)	.969 (.094)	.964 (.088)	.980 (.064)	.973 (.074)	.979 (.067)
**0.4per**	**NoSM**	.563 (.273)	.841 (.258)	.722 (.223)	.897 (.190)	.848 (.175)	.938 (.140)	.934 (.121)	.966 (.099)	.962 (.090)	.978 (.067)	.971 (.076)	.977 (.067)
**0.2per**	**NoSM**	.557 (.272)	.830 (.263)	.715 (.224)	.884 (.197)	.843 (.177)	.933 (.144)	.930 (.126)	.962 (.104)	.958 (.092)	.975 (.070)	.968 (.079)	.974 (.073)
**0.1per**	**NoSM**	.542 (.269)	.810 (.270)	.700 (.225)	.872 (.207)	.830 (.180)	.922 (.152)	.921 (.129)	.954 (.110)	.949 (.100)	.967 (.080)	.960 (.087)	.966 (.080)
**0.05per**	**NoSM**	.507 (.258)	.756 (.293)	.662 (.222)	.824 (.231)	.793 (.189)	.891 (.169)	.893 (.138)	.930 (.130)	.923 (.114)	.941 (.102)	.934 (.100)	.943 (.095)

MAF: minor allele frequency.

NoSin: no singletons.

NoAm: no ambiguous.

NoSM: no singletons or monomorphs.

0.05-3per: after removing SNPs with genotype missing rate higher than 0.05-3%.

The quality (information) scores were generated using IMPUTE2. The mean/average and standard deviation are shown outside and inside the brackets, respectively. We observed a high correlation between the imputed and study (true) genotypes, which incremented from low to high MAF ranges.

**Figure 5:**
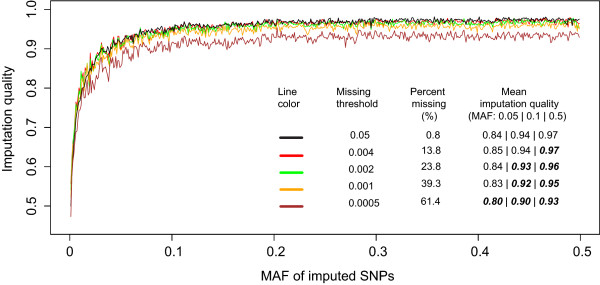
**Comparison of imputation quality of imputed SNPs.** The quality score columns list three SNP minor allele frequency (MAF) categories: very rare (0.001 < MAF < 0.05), rare (0.05 < MAF <0.1), and common (0.1 < MAF < 0.5). The results under the missing thresholds of 0.03 and 0.01 showed the similar patterns to those under the threshold of 0.05, and thus are not shown. Bold indicates *P* < 0.05 in the Welch two sample t-test between the missing rate of 0.05 (black line) and the other thresholds.

**Figure 6:**
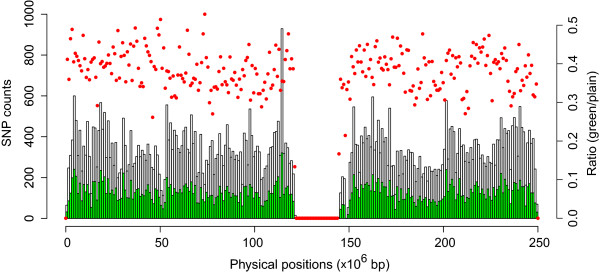
**Distribution of SNP missing genotypes.** The green histograms represent the numbers of remaining SNPs after removing the SNPs with missing rate > 0.05% while the plain histograms represent the total numbers of SNPs (on chromosome 1). The red circles represent the fractions of SNPs that passed the threshold. It is clear that the range of the fractions is narrow (i.e. 0.3-0.5).

Our results further showed that there was no noticeable effect on the imputation quality when the SNPs with genotype missing rate > 0.01 (667 SNPs) or 0.03 (939 SNPs) were excluded, regardless of the decrease of SNP density, when compared to the commonly-used genotype missing rate threshold of 0.05. This might be partially due to the fact that the assayed SNPs were of high quality, indicated by low genotype missing rates. For instance, the mean genotype missing rate was < 0.005 across all the SNPs with 0.001 < MAF < 0.5 on chromosome 1 (Additional file 3: Figure S2 and Additional file 4: Figure S3). We repeated the imputation procedures under new missing rate thresholds and measured their effects on imputation quality (Figure 5). The new thresholds included 0.004, 0.002, 0.001, and 0.0005, corresponding to the removals of 10,279 (13.8%), 17,785 (23.8%), 29,307 (39.3%), and 45,856 (61.4%) SNPs, respectively. Table 2 and Figure 5 show the comparisons of imputation quality measurements at the four missing thresholds across six different MAF ranges. As the missing threshold became more conservative (i.e. < 0.05), we observed a decrease in imputation quality where the higher MAFs exhibited more sensitivity to less stringent thresholds. For instance, the decrease emerged for the most common SNP group (0.1 < MAF < 0.5) at the missing threshold of 0.004, for the SNP group with 0.05 < MAF < 0.5 at the threshold of 0.002, and for the group containing rare SNPs (0.001 < MAF < 0.5) at the threshold of 0.0005. Surprisingly, we found that imputation of the rarest SNPs into genotyped genome regions tolerated very low SNP density (up to 39.3% lower when the missing threshold was 0.001) as long as the genotypes were of high quality (i.e. low missing rate). Moreover, exclusion of the SNPs with missing rate > 0.001 did not worsen imputation compared to the scenario where singletons were included in the reference (missing threshold = 0.05), particularly for SNPs with 0.001 < MAF < 0.005 (Additional file 5: Figure S4). Importantly, the locations of excluded SNPs (under the most conservative threshold) were distributed uniformly across the chromosome (Figure 6), indicating that the changes in imputation quality are very likely due to global, rather than local, changes in the SNP density of the genotype scaffold.

## Discussion

Both genome builds and allele definitions should be well-matched before combing or imputing one genotype data with another. In this study, we have developed a new, powerful, and user-friendly tool that can predict, and convert the genome builds and allele definitions simultaneously between multiple GWAS or deep sequencing genotype datasets for meta-analyses, imputations or both. Our GWAS data demonstrated the accuracy of predictions and performance of conversions. Our further imputations showed that the inclusion of singletons in the reference panel significantly decreased imputation quality. However, the exclusion of SNPs with missing rate > 0.001 led to comparably high imputation quality with the commonly-used threshold of 0.05 for rare SNPs (Table 2 and Figure 5 and Additional file 6: Figure S5), which implied that approximately 600,000 well-typed SNPs were likely to be sufficient for high quality genome-wide imputation of rare SNPs in our GWAS data.

### GACT pipeline

GACT achieved as low as 3.3% discarded genotypes (Table 1), which was significantly lower than commonly-observed mismatch rates. It should be noted that we always observe genotype mismatches in real datasets, particularly when one dataset is from microarray-based study and the other is from deep-sequencing-based study, like the case in Table 1. This is likely to be attributed to various factors, such as different experimental protocols, genotyping error rates, and disease statuses of research subjects. Interestingly, the genotype mismatch rates between different platforms are not significantly higher than those within same platforms. For instance, a recent study [13] showed 0.6-1.6% genotype mismatch rate within two deep-sequencing studies (Li et al’s data and the 1000 Genomes); by comparison, the 3.3% mismatch rate between two different platforms/samples is reasonably low. All these results demonstrated that it is required to correctly convert allele definitions prior to imputation or meta-analysis.

Table 3 shows the comparisons GACT with some of the existing tools that also include genome build and (or) allele definition conversion functions, including GWAMA [14], GenGen, METAL [15], and PLINK. The strengths of our tool include that it 1) can be easily connected to other commonly-used GWAS approaches (Figure 1); 2) can convert between any of the four commonly-used SNP allele definitions; 3) provides both the powerful command-line software and user-friendly web interface, where the latter can be easily used by biologists (no informatics training required except access to the internet); 4) can accurately predict allele definitions (and genome builds), which is particularly useful for investigators who use GWAS data from the dbGaP or other publicly available database; and 5) is computationally efficient, e.g., a typical conversion can be completed in a few seconds. In addition, the microarray-specific SNP definition information is used in GACT to flip the alleles and strands. Because it can convert data prior to association testing, meta-analysis and imputation, GACT complements existing tools and ensures allele definition and genome build consistency before using any of these tools. The limitation of our tool is that currently, the supported microarrays (primarily Illumina platforms) and genome-builds of the web version of GACT are not exhaustive (the command-line version has no such limitation; users can convert between any platforms and arrays using the command-line version of GACT). However, we will actively include conversions of other existing allele definitions, e.g., numerical alleles. We will provide continued scientific and technical support, and expand the list of arrays, genome builds, and new modules as new technologies and platforms become available.

**Table 3:** Comparisons of tools for genome build and allele definition conversions

**Complementary functionality**	**GenGen**	**GWAMA**	**METAL**	**PLINK**	**GACT**
Allele definition prediction	No	No	No	No	Yes
Uninformed strand/allele flip^1^	No	Yes	Yes	Yes	No
Informed allele conversion^2^	Yes^3^	No	No	No	Yes
Automatic allele conversion	Yes^3^	No	No	No^4^	Yes
Genome build prediction	No	No	No	No	Yes
Genome build conversion	No	No	No	Yes^4^	Yes
Command line	Yes	Yes	Yes	Yes	Yes
Interactive web interface	No	No	No	No	Yes

^1^“Uninformed” refers to flipping without SNP allele annotation knowledge.

^2^“Informed” refers to use of the original SNP definition and microarray-specific annotation information.

^3^GenGen converts between Top, Forward, A/B and 1/2 allele definitions; by comparison, GACT converts between Top, Forward, A/B and Plus definitions while the Plus definition is used by the 1000 Genomes Project and most next-generation sequencing studies.

^4^PLINK can strand- or allele-flip but it cannot directly convert from one allele definition to another, unless the user manually extracts information from the microarray annotation file; by comparison, GACT automatically converts between genome builds and allele definitions.

### Imputation after GACT Conversion

Imputation before combining GWAS datasets is desirable because of 1) increased power for identifying disease-associated variants, e.g. by more than 10% as suggested previously [16]; 2) higher SNP coverage for fine-mapping disease genes; 3) additional rare SNPs and applicability to other variants such as copy number variations or classical leukocyte antigen alleles [6]; and 4) cost- and time-efficiency compared with the molecular genotyping or sequencing experiments. Various studies have been carried out to evaluate or identify the factors that might affect imputation quality [12,17], including ambiguous, monomorphic, and singleton SNPs. Phasing of singletons is known to be challenging, and imputation becomes faster with no burden in the downstream association tests when singletons are removed from the reference. We found that, additionally, the removal of either ambiguous or monomorphic SNPs alone from the study data prior to phasing and imputation had no detectable effect on imputation. However, the exclusion of monomorphic and singleton SNPs from the reference increased imputation quality, which is in accordance with previous studies [12,17]. We further found that SNPs with very low MAF (0.001-0.005) showed the most significant increase of the imputation quality compared with the other MAF ranges (Table 2). This finding is important, particularly, for the rare variants, which are of increasing interest in the genetic studies of complex diseases and traits.

Balancing between genotype quality and genome coverage is important for imputation. The genotype missing thresholds of 0.05 to 0.02 [17] are generally recommended for quality controls in GWAS. However, no published studies have explicitly evaluated the effects of more conservative missing thresholds (than the commonly-used values) on imputation quality. Our assessments might provide a new perspective on the selection of genotype missing thresholds in imputation. Based on our GWAS data, an approximate number of 600 thousand well-typed SNPs are likely to be sufficient for high quality genome-wide imputation of rare SNPs (high quality assayed SNPs may compensate for low true-genotype density). However, further analyses are warranted to replicate the findings in additional arrays. It should be noted that only the data on chromosome 1 were used for most of the analyses based on our observation of similar genotype missing patterns or comparable results across all the chromosomes (Additional file 6: Figure S5 and Additional file 7: Figure S6).

## Conclusion

Ignorance of inconsistent allele definitions and genome builds or incorrect conversions lead to incorrect genetic association “findings”. In this study, we developed a comprehensive tool, GACT, with both powerful command-line and user-friendly web interface versions to predict, and convert both genome builds and allele definitions between multiple GWAS (or deep sequencing) genotype data, which is required for all imputations and genome-wide meta-analyses. GACT will facilitate and ease a broad use of the GWAS data from the dbGaP and other publicly available genotype repositories for large-scale secondary analyses and multi-laboratory collaborations in the genetic association studies of human diseases.

## Availability and requirements

**Project name:** GACT: **G**enome build and **A**llele definition **C**onversion **T**ool

**Project homepage:**http://www.uvm.edu/genomics/software/gact

**Operating system(s):** Linux, UNIX (for command version) and Windows (for interactive web version)

**Programming language:** Python, Ruby, Hypertext Preprocessor (PHP), and Bash scripts

**License:** GPL-3

**Availability:** GACT (both command-line and web versions), including source code, documentation, and examples, is freely available for non-commercial use with no restrictions at http://www.uvm.edu/genomics/software/gact and http://asulovar.w3.uvm.edu/gact.

## Competing interests

The authors declare that they have no competing interests.

## Authors’ contributions

DL conceived, organized and supervised the project. AS wrote the source code and conducted the analyses. AS and DL drafted the manuscript. Both authors read and approved the final manuscript.

## Supplementary Material

Additional file 1: Table S1 Comparison of imputation quality before and after genotype conversion using GACT. Click here for file

Additional file 2: Figure S1 The feed-forward backpropagation neural network. The 3 input neurons correspond to the proportion of CT, TC and GA. The number in black next to each edge represents the weight of that edge. The numbers in blue represent the activation threshold for each hidden node, as defined by the activation function of the neural network, after training. There were three such networks in GACT, where each was trained to make an independent prediction on the likelihood that the input map file was using one of the three allele definitions: Plus (using the 1000 Genomes), Forward (using dbSNP) and Top (using our GWAS data). The artificial neural network that generated the largest likelihood determined the final allele definition. The A/B definition, which can be distinguished directly, was not included in the network. Click here for file

Additional file 3: Figure S2 Imputation quality and genotype missing rate across allele frequencies. The missing frequency measurement is the average of missing genotype rates for all the SNPs at a given MAF. The numbers of the SNPs that were excluded were 45,856, 29,307, 17,785, 10,279, 4,667, and 939 (out of 74,638) when the genotype missing rate thresholds were set at 0.0005, 0.001, 0.002, 0.004, 0.01, and 0.03, respectively. The red curve shows the information (quality) scores of the imputed genotypes across the full allele frequency range (0–1). The green histogram shows the genotype missing rate distribution across the full range of MAFs (0–0.5) under the missing genotype threshold of 0.05. The MAF scale (0–0.5) was adopted, instead of a full scale (0–1), based on our autocorrelation analyses of the imputation quality curves which showed that the head-10% and tail-10% were significantly correlated (Additional file 3: Figure S2). Other chromosome showed the similar patterns, and thus only the results of chromosome 1 are shown. Click here for file

Additional file 4: Figure S3 Autocorrelation plots of mean imputation scores. This figure corresponds to the full range of allele frequencies that is shown in Additional file 2: Figure S1 (red line). The Lag axis represents the shift of the data points, one number at a time at a rate of 0.001, while the ACF axis represents an adjusted correlation factor between the “shifted” data and the original data. The histograms outside of the dotted blue lines represent the regions with higher correlation than expected by chance alone (at confidence level > 95%). Moreover, this autocorrelation plot indicated that the regions of allele frequency < 0.1 and > 0.9 were significantly correlated at the confidence level of > 0.95. Based on this result we combined both the upper and lower halves to generate MAFs (0–0.5), instead of the full range of allele frequencies (0–1). Click here for file

Additional file 5: Figure S4 Changes of imputation quality across different genotype missing thresholds. When singleton and monomorphic sites were excluded from the reference, the highest imputation quality was achieved compared to other scenarios. When the entire reference was used, the imputation quality was particularly low for very rare SNPs (0.001 < MAF < 0.005; red line). The less rare and common SNPs (MAF > 0.005, i.e., green, blue, orange, yellow, and black lines) were not influenced as much by the removal of singletons and monomorphs in reference panel. Moreover, for very rare SNPs the exclusion of as many as 39.3% of the SNPs (i.e., “0.1per_NoSM” in the figure) led to a smaller decrease of imputation quality than inclusion of singletons and monomorphic SNPs in reference panel. NoSin: no reference singletons; NoAm: no reference ambiguous SNPs; NoSM: no reference singletons or monomorphs; *per: after removing study SNPs with genotype missing rate higher than *%. Click here for file

Additional file 6: Figure S5 Imputation quality versus missing threshold across 21 autosomes. The green histograms represent genotype missing levels for SNPs that are measured using MAFs from 0.001 to 0.5 while the red curves represent imputation qualities for SNPs that are measured using the full allele frequency from 0.001 to 1. Click here for file

Additional file 7: Figure S6 Pearson correlations of mean imputation quality scores between the MAF windows of 0–0.1 and 0.9-1.0. The plots show that the head 10% of the imputation curves is correlated with its tail 10% for all chromosomes, suggesting it is necessary to convert the allele frequencies of imputed SNPs from the range of 0.001-1 to range of 0.001-0.5. Click here for file
