# Supplementary material for: GACT: a Genome build and Allele definition Conversion Tool for SNP imputation and meta-analysis in genetic association studies
Source: BMC Genomics. 2014 Jul 19;15:610. doi: 10.1186/1471-2164-15-610 (PMC4223508; doi:10.1186/1471-2164-15-610)
Supplement: Additional file 1: Table S1 — Comparison of imputation quality before and after genotype conversion using GACT. [file 1471-2164-15-610-S1.doc]

| **MAFs** | **Before** | **After** |
| --- | --- | --- |
| 0.001-0.005 | 0.56 (.30) | 0.57 (.30) |
| 0.005-0.01 | 0.72 (.22) | 0.73 (.22) |
| 0.01-0.05 | 0.84 (.18) | 0.85 (.17) |
| 0.05-0.1 | 0.93 (.12) | 0.94 (.12) |
| 0.1-0.3 | 0.96 (.09) | 0.97 (.09) |
| 0.3-0.5 | 0.97 (.08) | 0.98 (.07) |

Imputation is the process of using a reference haplotype panel at a dense set of SNPs (i.e., the 1000 Genomes Project) to impute into a sample of individuals genotyped for a subset of these SNPs (i.e., the GWAS data). The numbers in this table represent the mean imputation quality scores after the basic quality control of removing SNPs with missing genotype rate > 0.05. The standard deviations are shown in brackets. Imputing into less dense SNP regions (i.e. before GACT conversion) revealed lower imputation scores than denser SNP regions (i.e. after GACT conversion). This table shows the increase (improvement) of imputation quality based on our GWAS data (“Forward/Reverse”) and the 1000 Genomes data (“Plus/Minus”). However, it should be noted that the improvement would be much *higher* if data with the “TOP/BOT” definition were used since the mismatch rate between the “TOP/BOT” and “Plus/Minus” definitions was larger (Table 1). Other chromosome showed similar patterns, and thus only the results of chromosome 1 are shown.
