# Supplementary figures and images for: GACT: a Genome build and Allele definition Conversion Tool for SNP imputation and meta-analysis in genetic association studies
Source: BMC Genomics. 2014 Jul 19;15:610. doi: 10.1186/1471-2164-15-610 (PMC4223508; doi:10.1186/1471-2164-15-610)

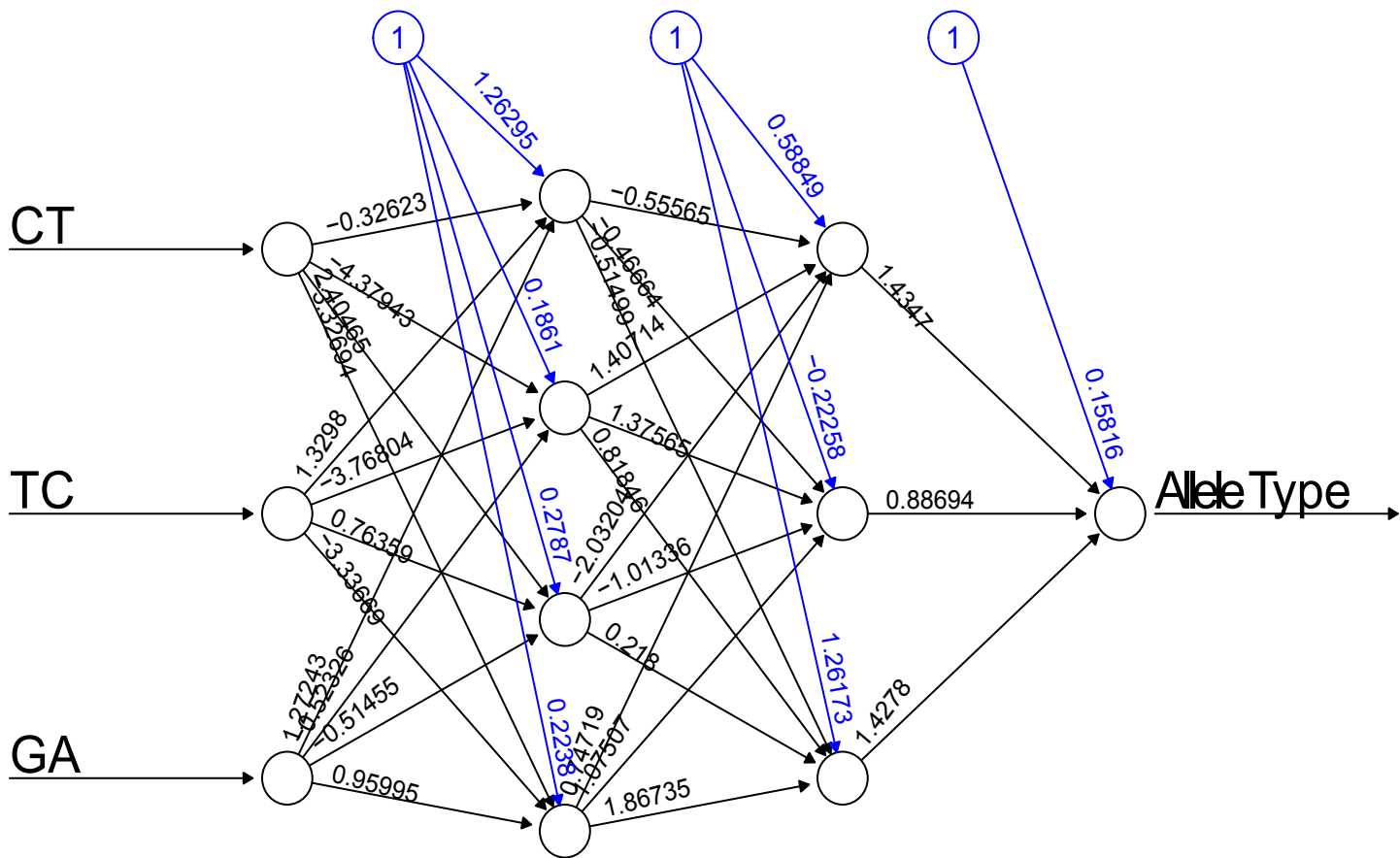

Supplement: Additional file 2: Figure S1 — The feed-forward backpropagation neural network. The 3 input neurons correspond to the proportion of CT, TC and GA. The number in black next to each edge represents the weight of that edge. The numbers in blue represent the activation threshold for each hidden node, as defined by the activation function of the neural network, after training. There were three such networks in GACT, where each was trained to make an independent prediction on the likelihood that the input map file was using one of the three allele definitions: Plus (using the 1000 Genomes), Forward (using dbSNP) and Top (using our GWAS data). The artificial neural network that generated the largest likelihood determined the final allele definition. The A/B definition, which can be distinguished directly, was not included in the network. [file 1471-2164-15-610-S2.pdf]

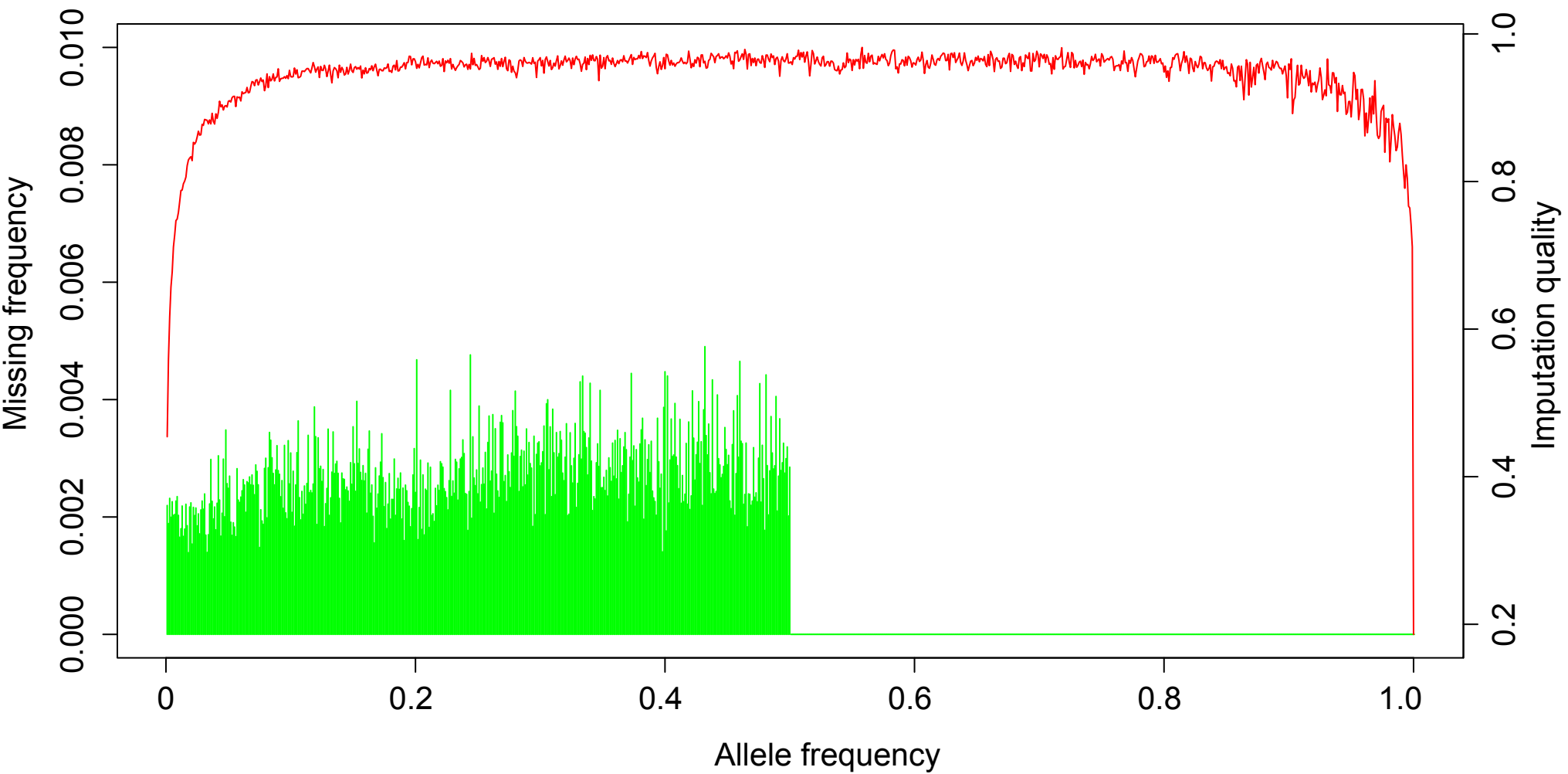

Supplement: Additional file 3: Figure S2 — Imputation quality and genotype missing rate across allele frequencies. The missing frequency measurement is the average of missing genotype rates for all the SNPs at a given MAF. The numbers of the SNPs that were excluded were 45,856, 29,307, 17,785, 10,279, 4,667, and 939 (out of 74,638) when the genotype missing rate thresholds were set at 0.0005, 0.001, 0.002, 0.004, 0.01, and 0.03, respectively. The red curve shows the information (quality) scores of the imputed genotypes across the full allele frequency range (0–1). The green histogram shows the genotype missing rate distribution across the full range of MAFs (0–0.5) under the missing genotype threshold of 0.05. The MAF scale (0–0.5) was adopted, instead of a full scale (0–1), based on our autocorrelation analyses of the imputation quality curves which showed that the head-10% and tail-10% were significantly correlated (Additional file 3: Figure S2). Other chromosome showed the similar patterns, and thus only the results of chromosome 1 are shown. [file 1471-2164-15-610-S3.pdf]

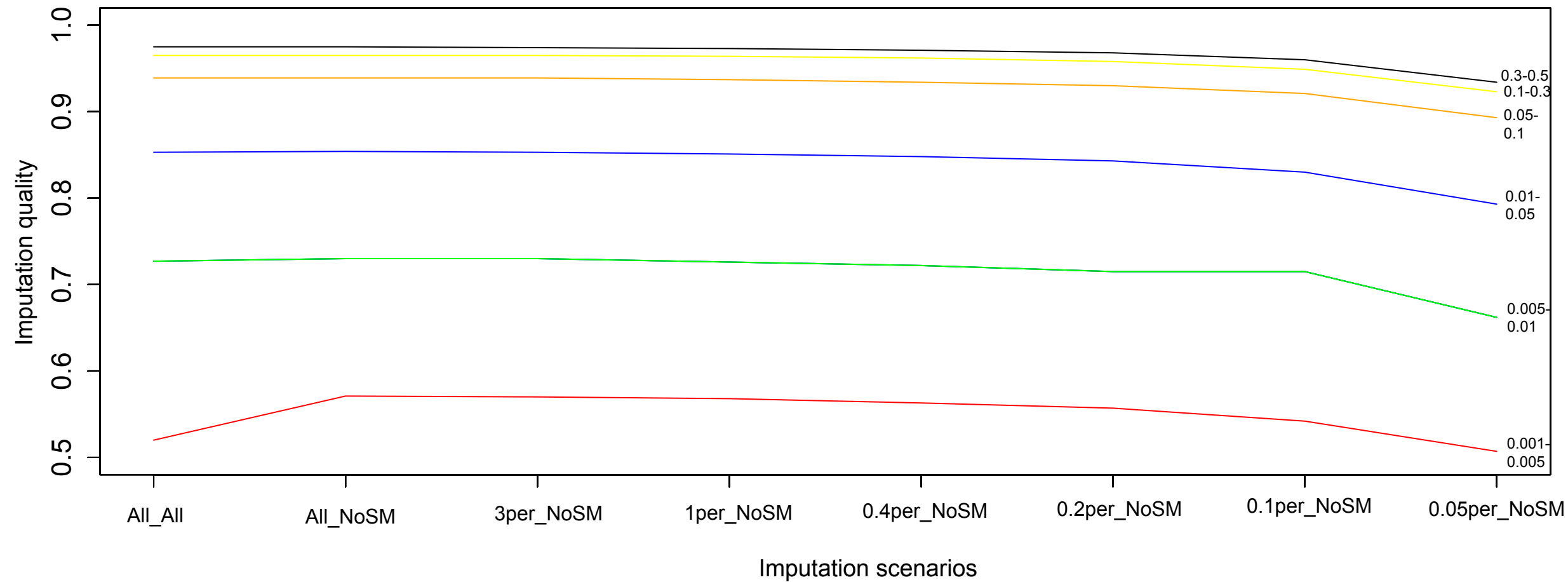

Supplement: Additional file 5: Figure S4 — Changes of imputation quality across different genotype missing thresholds. When singleton and monomorphic sites were excluded from the reference, the highest imputation quality was achieved compared to other scenarios. When the entire reference was used, the imputation quality was particularly low for very rare SNPs (0.001 < MAF < 0.005; red line). The less rare and common SNPs (MAF > 0.005, i.e., green, blue, orange, yellow, and black lines) were not influenced as much by the removal of singletons and monomorphs in reference panel. Moreover, for very rare SNPs the exclusion of as many as 39.3% of the SNPs (i.e., “0.1per_NoSM” in the figure) led to a smaller decrease of imputation quality than inclusion of singletons and monomorphic SNPs in reference panel. NoSin: no reference singletons; NoAm: no reference ambiguous SNPs; NoSM: no reference singletons or monomorphs; *per: after removing study SNPs with genotype missing rate higher than *%. [file 1471-2164-15-610-S5.pdf]

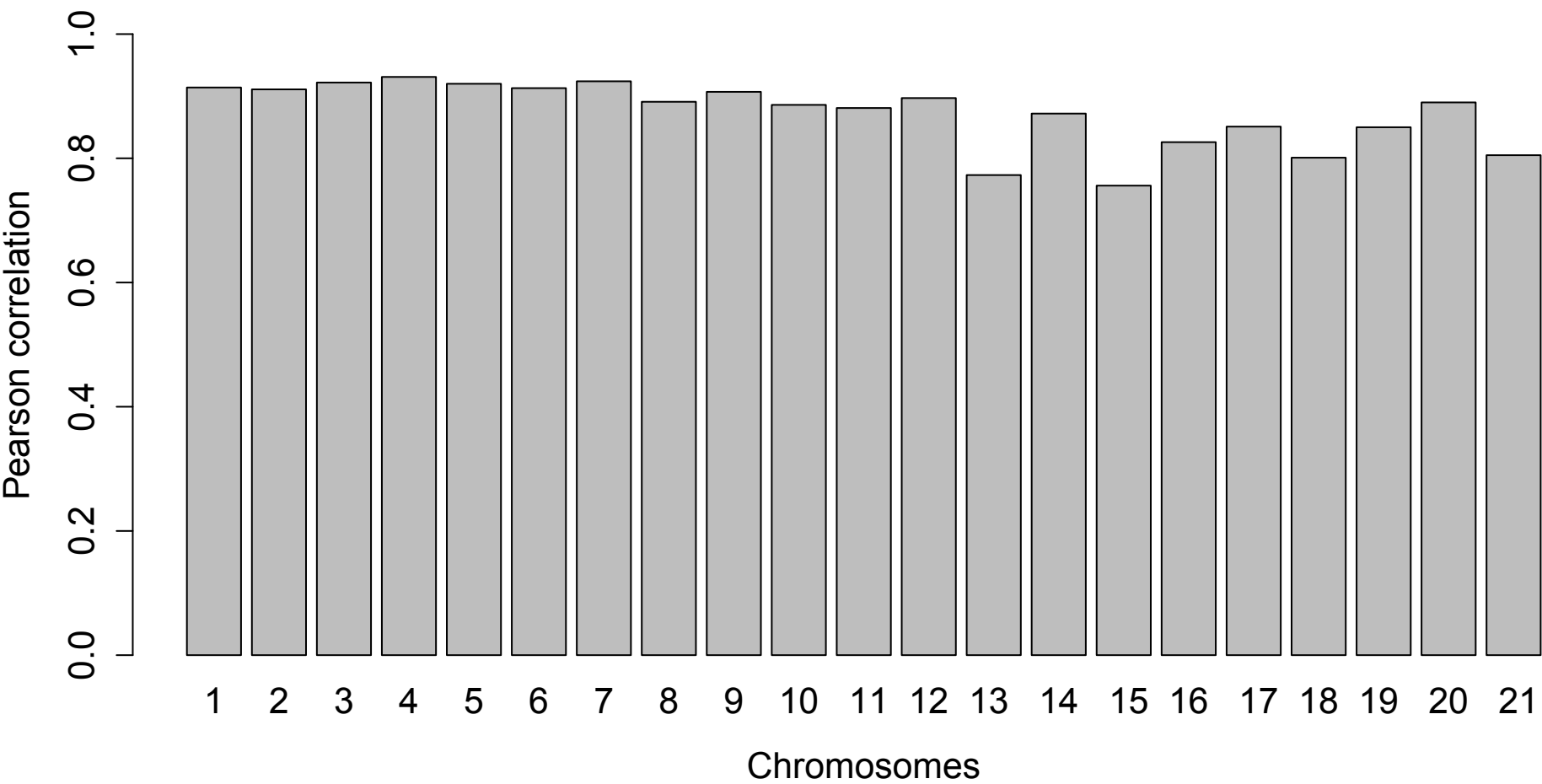

Supplement: Additional file 7: Figure S6 — Pearson correlations of mean imputation quality scores between the MAF windows of 0–0.1 and 0.9-1.0. The plots show that the head 10% of the imputation curves is correlated with its tail 10% for all chromosomes, suggesting it is necessary to convert the allele frequencies of imputed SNPs from the range of 0.001-1 to range of 0.001-0.5. [file 1471-2164-15-610-S7.pdf]
