# Supplementary material for: GACT: a Genome build and Allele definition Conversion Tool for SNP imputation and meta-analysis in genetic association studies
Source: BMC Genomics. 2014 Jul 19;15:610. doi: 10.1186/1471-2164-15-610 (PMC4223508; doi:10.1186/1471-2164-15-610)
Supplement: Additional file 4: Figure S3 — Autocorrelation plots of mean imputation scores. This figure corresponds to the full range of allele frequencies that is shown in Additional file 2: Figure S1 (red line). The Lag axis represents the shift of the data points, one number at a time at a rate of 0.001, while the ACF axis represents an adjusted correlation factor between the “shifted” data and the original data. The histograms outside of the dotted blue lines represent the regions with higher correlation than expected by chance alone (at confidence level > 95%). Moreover, this autocorrelation plot indicated that the regions of allele frequency < 0.1 and > 0.9 were significantly correlated at the confidence level of > 0.95. Based on this result we combined both the upper and lower halves to generate MAFs (0–0.5), instead of the full range of allele frequencies (0–1). [file 1471-2164-15-610-S4.pdf]

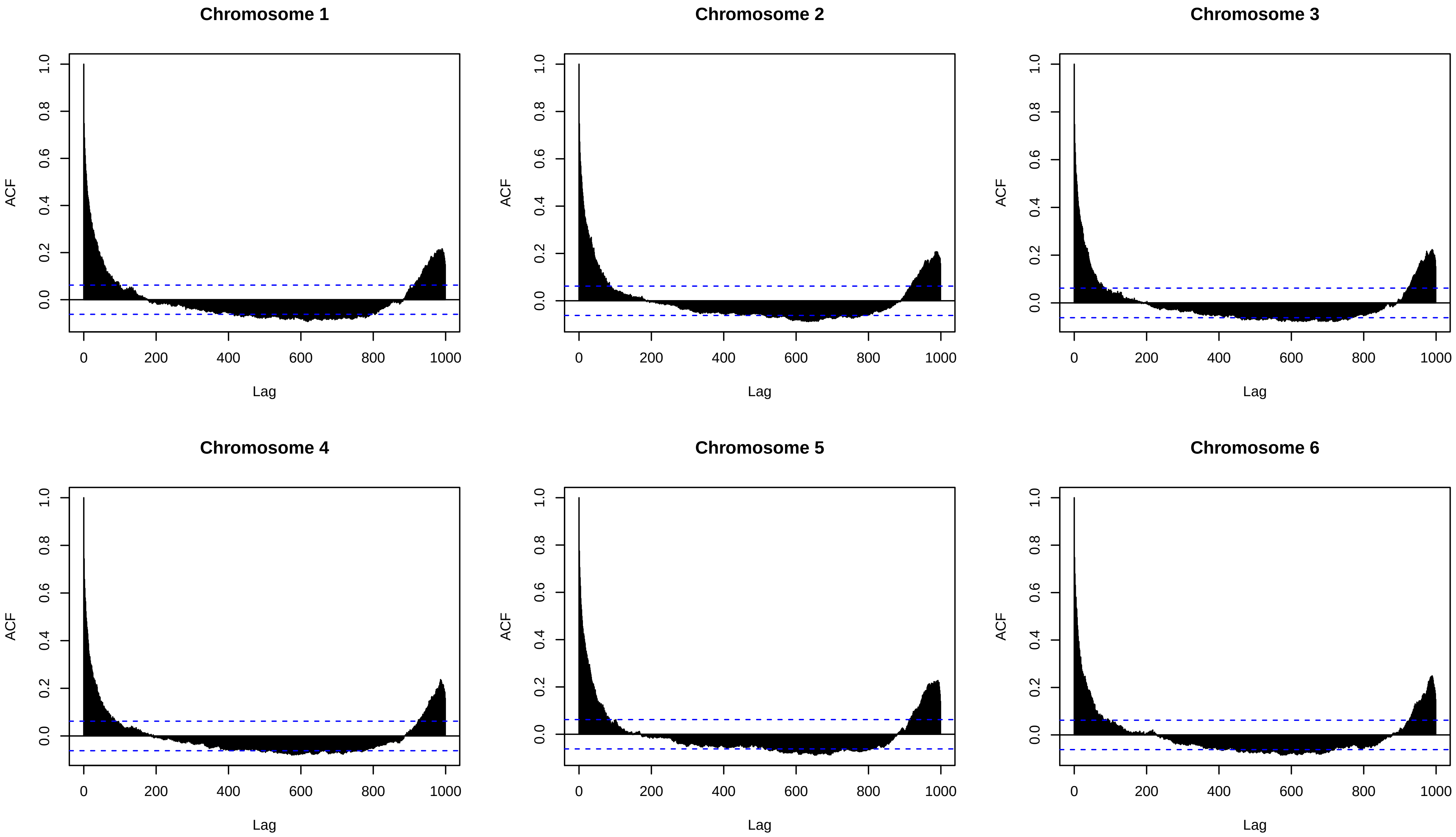
Chromosome 1
Chromosome 2
Chromosome 3
Chromosome 4
Chromosome 5
Chromosome 6
ACF
Lag
0.0
0.2
0.4
0.6
0.8
1.0
0
200
400
600
800
1000

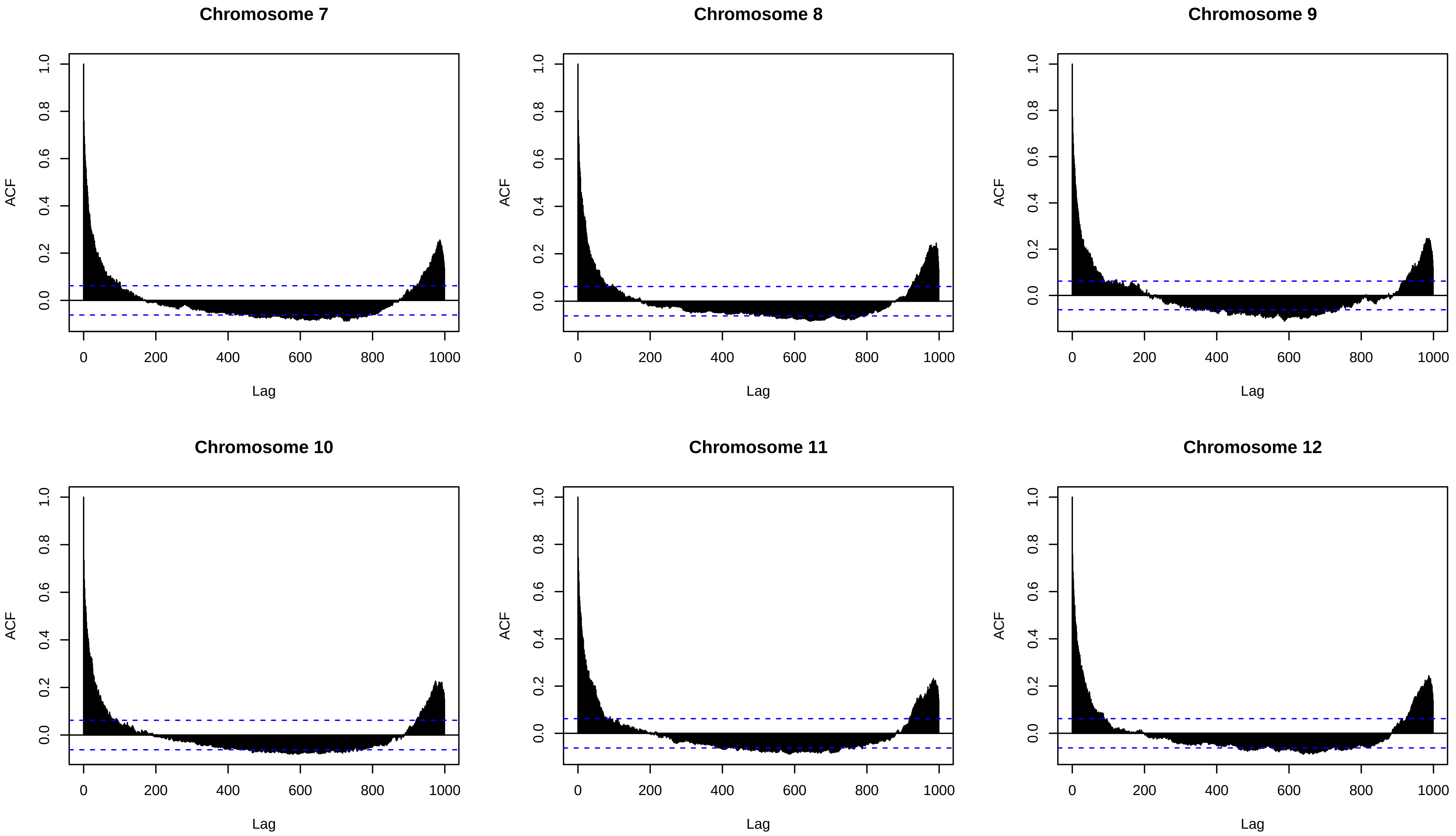
Chromosome 7
Chromosome 8
Chromosome 9
Chromosome 10
Chromosome 11
Chromosome 12
ACF
Lag
0.0
0.2
0.4
0.6
0.8
1.0
0
200
400
600
800
1000

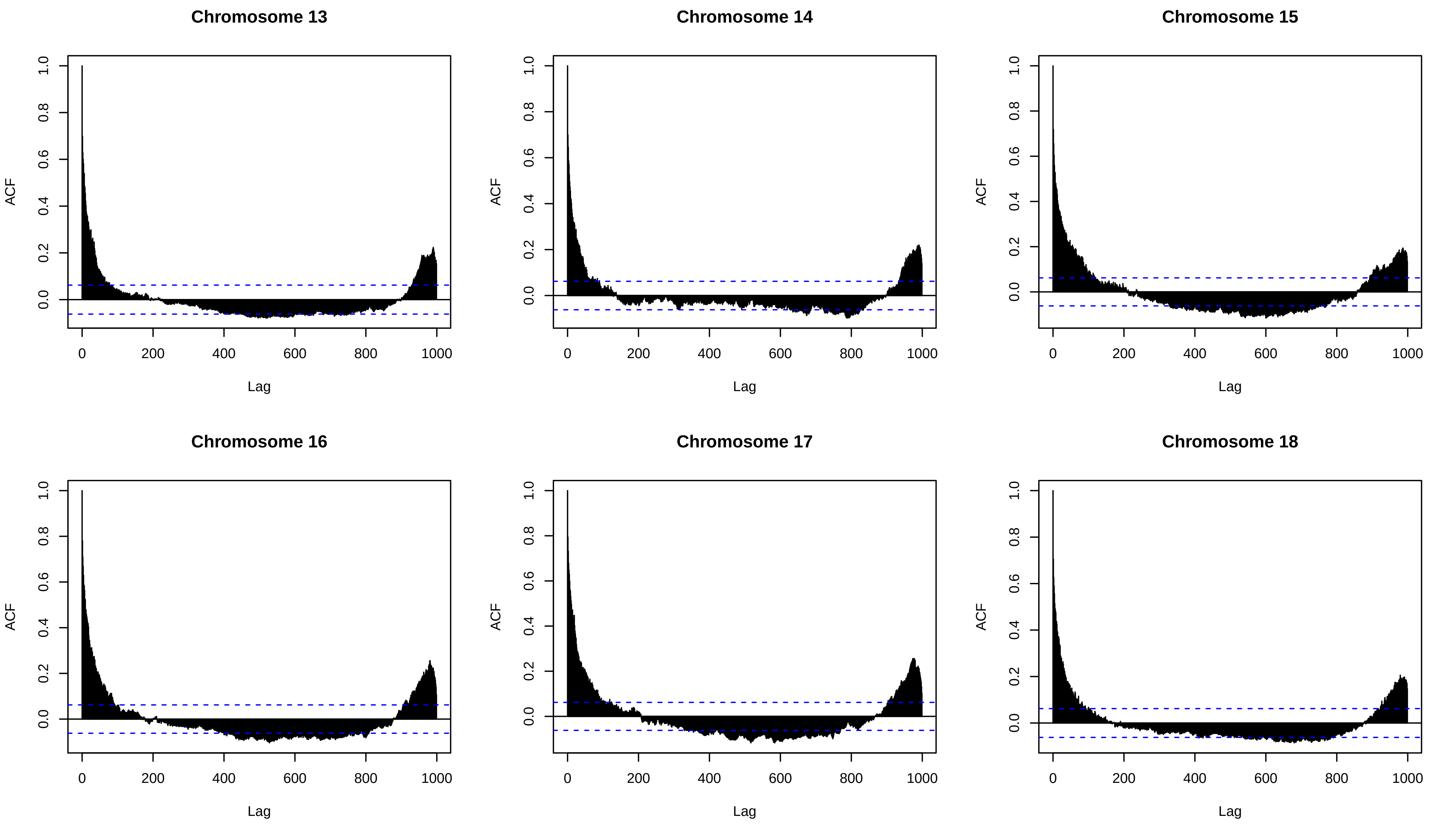
Chromosome 13
Chromosome 14
Chromosome 15
Chromosome 16
Chromosome 17
Chromosome 18
ACF
Lag
0.0
0.2
0.4
0.6
0.8
1.0
0
200
400
600
800
1000

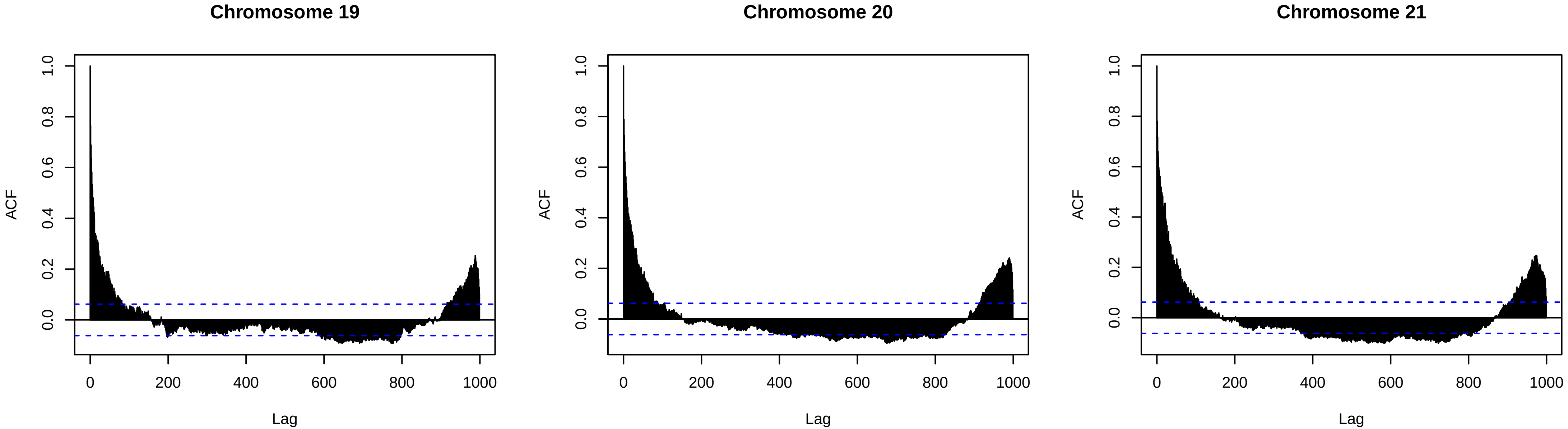
Chromosome 19
Chromosome 20
Chromosome 21
ACF
Lag
0.0
0.2
0.4
0.6
0.8
1.0
0
200
400
600
800
1000
