# Supplementary material for: GACT: a Genome build and Allele definition Conversion Tool for SNP imputation and meta-analysis in genetic association studies
Source: BMC Genomics. 2014 Jul 19;15:610. doi: 10.1186/1471-2164-15-610 (PMC4223508; doi:10.1186/1471-2164-15-610)
Supplement: Additional file 6: Figure S5 — Imputation quality versus missing threshold across 21 autosomes. The green histograms represent genotype missing levels for SNPs that are measured using MAFs from 0.001 to 0.5 while the red curves represent imputation qualities for SNPs that are measured using the full allele frequency from 0.001 to 1. [file 1471-2164-15-610-S6.pdf]

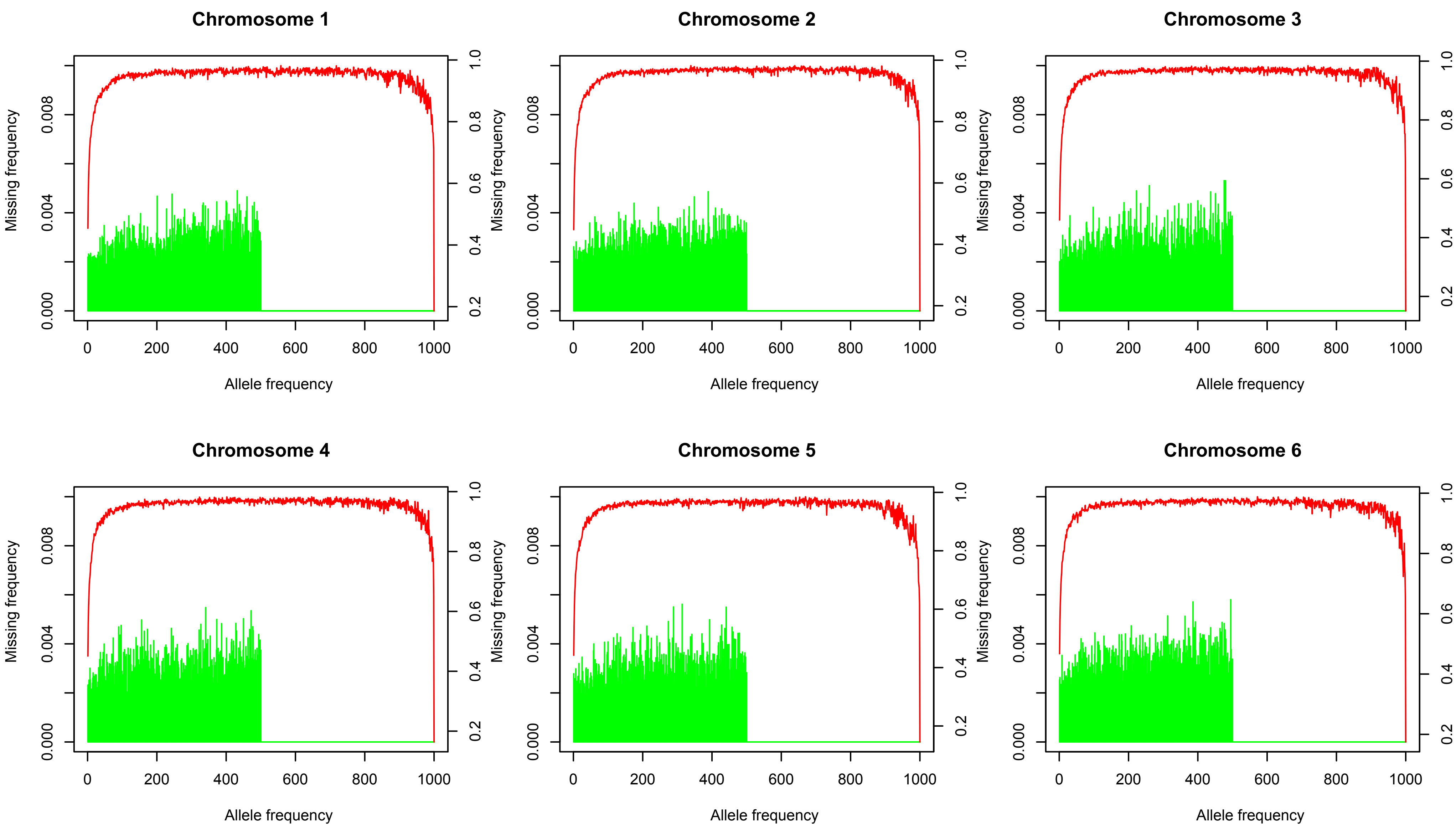

Chromosome 1
Chromosome 2
Chromosome 3
Chromosome 4
Chromosome 5
Chromosome 6
Missing frequency
Allele frequency
0.000
0.004
0.008
0.2
0.4
0.6
0.8
1.0
0
200
400
600
800
1000

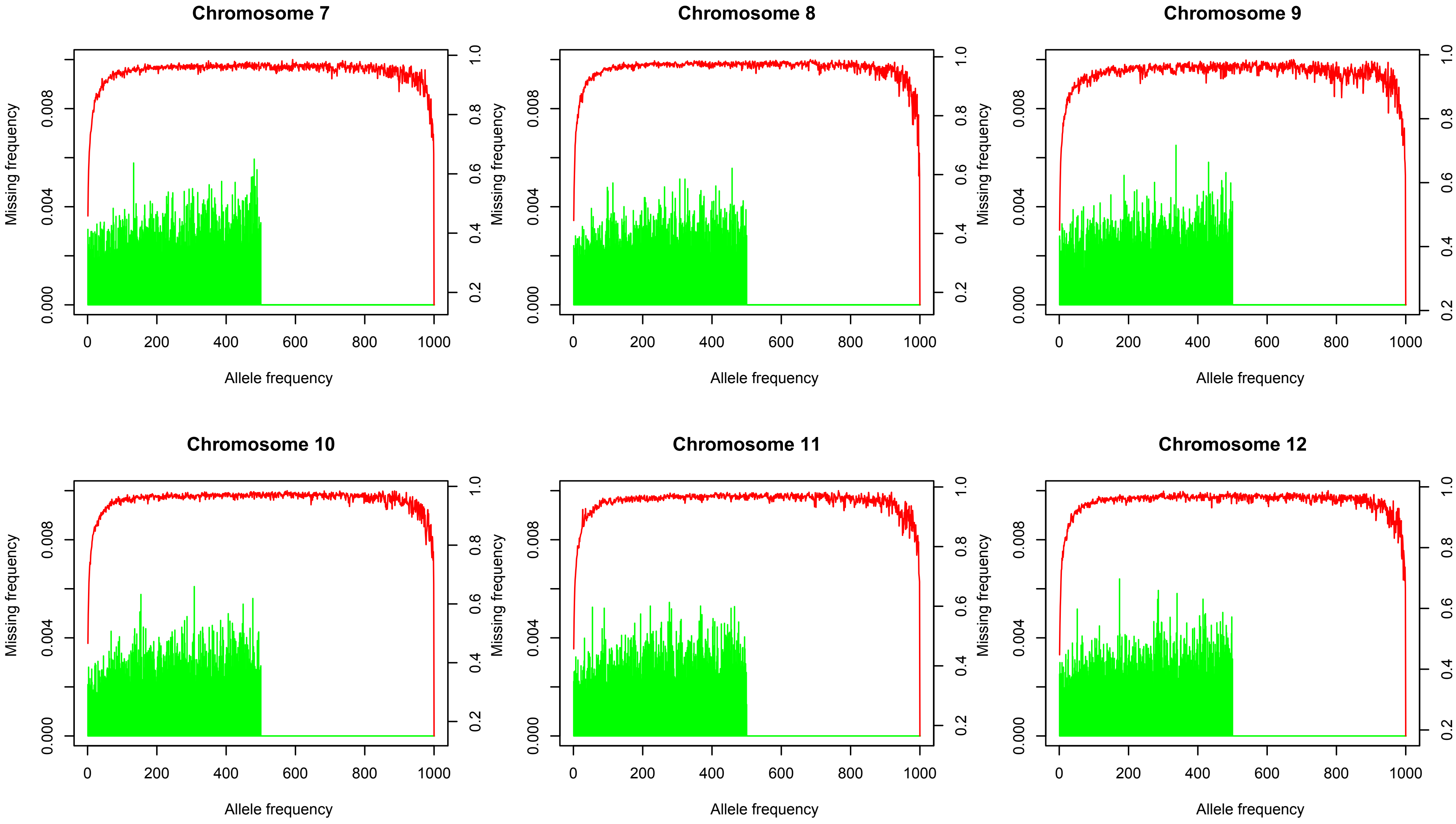

Chromosome 7
Chromosome 8
Chromosome 9
Chromosome 10
Chromosome 11
Chromosome 12
Missing frequency
Allele frequency
0.000
0.004
0.008
0.2
0.4
0.6
0.8
1.0
0
200
400
600
800
1000

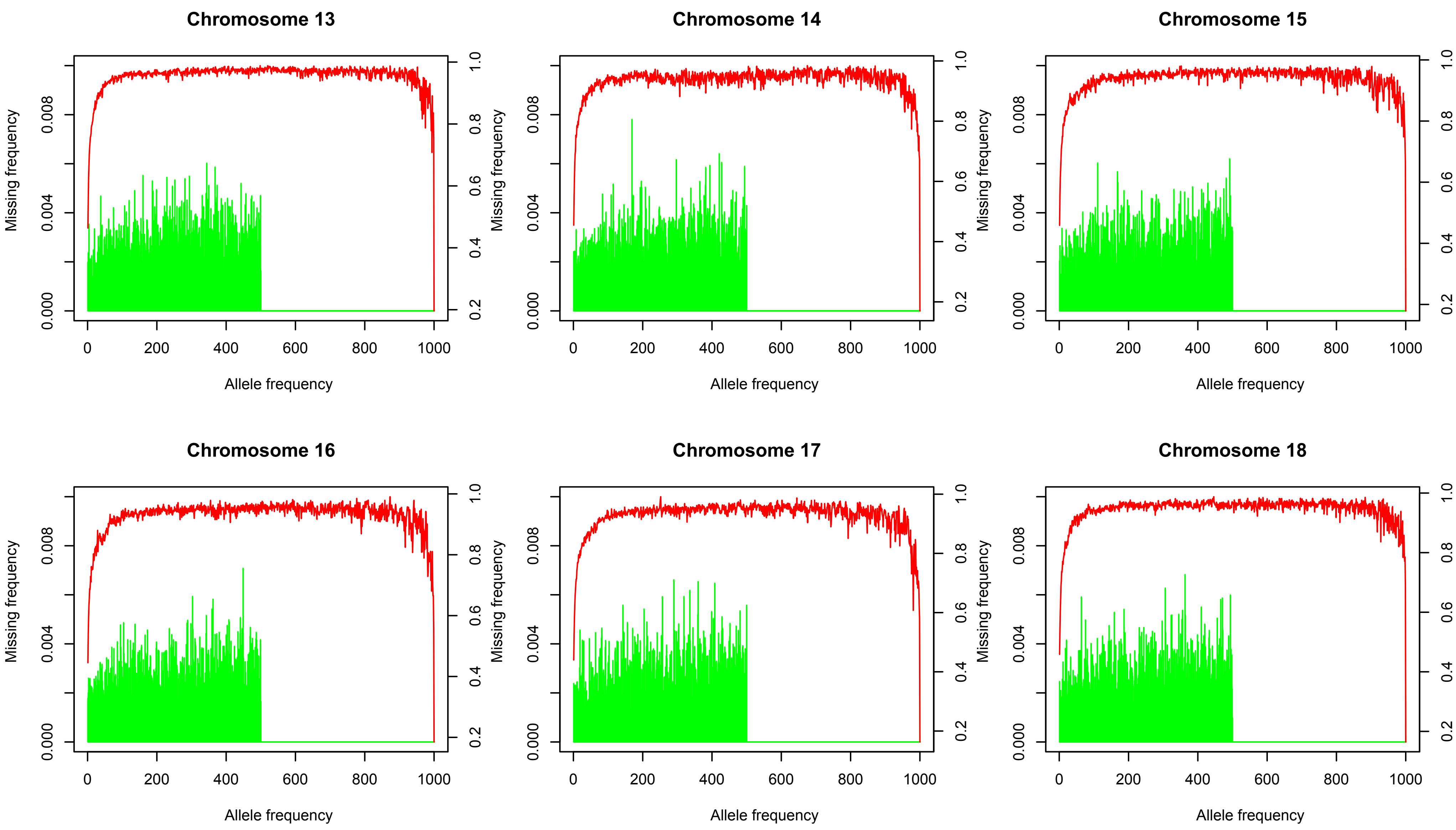
Chromosome 13
Chromosome 14
Chromosome 15
Chromosome 16
Chromosome 17
Chromosome 18
Missing frequency
Allele frequency
0.000
0.004
0.008
0.2
0.4
0.6
0.8
1.0
0
200
400
600
800
1000

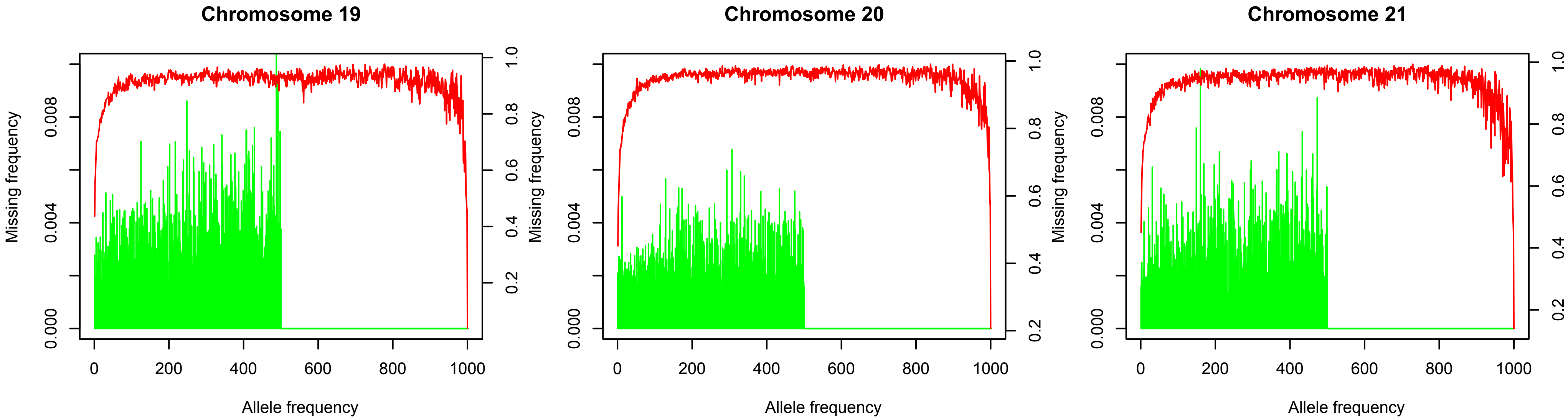
Chromosome 19
Chromosome 20
Chromosome 21
Missing frequency
0.000
0.004
0.008
0.2
0.4
0.6
0.8
1.0
0
200
400
600
800
1000
Allele frequency
